# Occupancy versus colonization–extinction models for projecting population trends at different spatial scales

**DOI:** 10.1002/ece3.6124

**Published:** 2020-03-03

**Authors:** Jenni Nordén, Philip J. Harrison, Louise Mair, Juha Siitonen, Anders Lundström, Oskar Kindvall, Tord Snäll

**Affiliations:** ^1^ Norwegian Institute for Nature Research Oslo Norway; ^2^ Artdatabanken Swedish University of Agricultural Sciences (SLU) Uppsala Sweden; ^3^ Natural Resources Institute Finland (Luke) Helsinki Finland; ^4^ Department of Forest Resource Management Swedish University of Agricultural Sciences (SLU) Umeå Sweden; ^5^ Calluna AB Göteborg Sweden; ^6^ Present address: Department of Pharmaceutical Biosciences Uppsala University Uppsala Sweden; ^7^ Present address: School of Natural and Environmental Sciences Newcastle University Newcastle upon Tyne UK

**Keywords:** data resolution, environmental driver, population dynamics, predictive modeling, scenario, spatial modeling scale

## Abstract

Understanding spatiotemporal population trends and their drivers is a key aim in population ecology. We further need to be able to predict how the dynamics and sizes of populations are affected in the long term by changing landscapes and climate. However, predictions of future population trends are sensitive to a range of modeling assumptions. Deadwood‐dependent fungi are an excellent system for testing the performance of different predictive models of sessile species as these species have different rarity and spatial population dynamics, the populations are structured at different spatial scales, and they utilize distinct substrates. We tested how the projected large‐scale occupancies of species with differing landscape‐scale occupancies are affected over the coming century by different modeling assumptions. We compared projections based on occupancy models against colonization–extinction models, conducting the modeling at alternative spatial scales and using fine‐ or coarse‐resolution deadwood data. We also tested effects of key explanatory variables on species occurrence and colonization–extinction dynamics. The hierarchical Bayesian models applied were fitted to an extensive repeated survey of deadwood and fungi at 174 patches. We projected higher occurrence probabilities and more positive trends using the occupancy models compared to the colonization–extinction models, with greater difference for the species with lower occupancy, colonization rate, and colonization:extinction ratio than for the species with higher estimates of these statistics. The magnitude of future increase in occupancy depended strongly on the spatial modeling scale and resource resolution. We encourage using colonization–extinction models over occupancy models, modeling the process at the finest resource‐unit resolution that is utilizable by the species, and conducting projections for the same spatial scale and resource resolution at which the model fitting is conducted. Further, the models applied should include key variables driving the metapopulation dynamics, such as the availability of suitable resource units, habitat quality, and spatial connectivity.

## Graphical abstract

We show that the projected future trends of fungal populations are sensitive to type of modeling performed (occupancy patterns vs. colonization–extinction dynamics), the rarity of the species, the spatial scale of model fitting, and the resource‐unit data resolution. Our conclusions are based on Bayesian modeling of an extensive resurvey dataset.

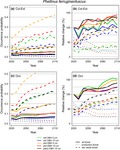

## 1 INTRODUCTION

Understanding spatial and temporal population trends and the drivers behind them is a key aim in population ecology (Turchin, 2003). Such knowledge is also necessary when planning actions to mitigate the pervasive effects of habitat loss, fragmentation, and climate change (Pressey, Cabeza, Watts, Cowling, & Wilson, 2007). In fragmented landscapes, large‐scale population trends often result from metapopulation dynamics, through the local processes of colonization and extinction (Hanski, 1999). Theoretical studies suggest that metapopulation viability strongly depends on landscape features and processes such as the availability, size and longevity of suitable habitat patches (e.g., old stands or appropriate tree structures in forest landscapes), spatial connectivity, patterns of patch destruction and creation, and interactions between these (Johst et al., 2011).

Habitat patches naturally appear and disappear through succession and disturbance, but in production landscapes, these processes are largely replaced by management and conservation actions (Kuuluvainen, 2009). A continuous local supply of new resource units is critical for the persistence of species that are confined to ephemeral resource units such as living or dead trees. These species need to balance the local extinctions (stochastic or resulting from resource‐unit disappearance) with local colonizations of new resource units. These units need to have high enough density in space and frequency through time to allow regional persistence (Gourbiere & Gourbiere, 2002; Snäll, Ribeiro, & Rydin, 2003).

It is important that forecasts of the long‐term effects of management and conservation actions on species populations are realistic and accurate, because today's decisions may give rise to adverse or unexpected consequences that may be difficult to overturn (Guisan et al., 2013). To parameterize models of spatially realistic metapopulation dynamics (Hanski, 1999) to be used as a basis of forecasts, one should ideally have collected data repeatedly on the size and distribution of all habitat patches and local populations, and information about the dispersal rate and range of the species (Higgins & Cain, 2002). Such data are usually lacking, and thus, other solutions must be sought.

A common method to predict species responses to future environmental changes is to use species distribution models (SDMs) fit to a single (static) snapshot of presence/absence data across the landscape (Elith & Leathwick, 2009). These include occupancy models which we evaluate for projection herein. SDMs associate the spatial pattern of a species' occurrence across a subset of the populations in the landscape with habitat and climate data. Such models fitted to snapshot pattern data, however, assume that the current occurrence pattern of the species is at metapopulation equilibrium with its environment. Violations of this assumption can produce biased results as at disequilibrium, occupancy–environment relationship is expected to vary over time and space (Yackulic, Nichols, Reid, & Der, 2015). For species with high colonization–extinction dynamics, for example, many mammals, birds, and insects, the species distribution pattern can indeed be assumed to much depend on the current landscape structure (Ovaskainen & Hanski, 2002). If the landscape structure changes, for example, due to management operations, the species distribution will promptly adjust to the new structure. For such species, SDMs may produce reliable projections of future population trends. In contrast, for sessile species with slow colonization–extinction dynamics, such as probably many fungi and plants, the distribution patterns may not reflect the present spatial structure of the landscape (Ovaskainen & Hanski, 2002). With changing landscape structure, the species distribution patterns will reflect the past rather than the current landscape structure (Paltto, Nordén, Götmark, & Franc, 2006; Snäll, Hagström, Rudolphi, & Rydin, 2004). Thus for sessile species, a SDM may be inappropriate for predictive modeling, for example, resulting in overly optimistic projections in situations where the area and connectivity of the habitat have decreased over time.

When data are available over multiple time points, it is preferable to acknowledge the temporal change and model the processes which generated the patterns (Gimenez et al., 2014), for instance using what we refer here to as colonization–extinction models (also known as dynamic occupancy models, occupancy dynamics models, or multiseason occupancy models) (MacKenzie, Nichols, Hines, Knutson, & Franklin, 2003). Under models for colonization–extinction dynamics, the past landscape structure becomes less influential, because colonization events that take place between the two surveys reflect the current locations of the dispersal sources. Especially for species with slow colonization–extinction dynamics, SDMs based on occupancy–environment relationships can be expected to produce biased future occupancy patterns (Ovaskainen & Hanski, 2002), and it should be better to base predictions on models that incorporate both rates of local colonization and extinction and their dependence on environmental conditions (Yackulic et al., 2015). Projections of future population development have focused on changes in the distribution patterns (del Rosario Avalos & Hernandez, 2015), while estimates of the future summed occupancies or population sizes have to date received little attention.

A major issue in predictive ecology is the scale at which ecological processes should be considered (Chave, 2013; Evans et al., 2013; Mouquet et al., 2015). Predictions made from models fit to data at different spatial modeling scales can lead to drastically different conclusions (León‐Cortés, Cowley, & Thomas, 1999). When modeling is performed at too large a spatial modeling scale, local heterogeneities in resource quality and quantity relevant for the species in question will go undetected (Mouquet et al., 2015). SDM model performance has been shown to depend on the chosen grain size, especially for systems that can be relatively accurately modeled, but the direction and strength of this effect depend strongly on the type of species (Guisan et al., 2007).

In studies of species that are restricted to a particular resource unit in the habitat patch, such as living trees or deadwood, field surveys often involve a trade‐off between resource resolution, that is, the minimum size or the types of the resource unit to be included (e.g., minimum deadwood diameter), and the survey area covered (Zotz & Bader, 2011). If small or particular kinds of resource units are abundant, including them in the survey may make it difficult to attain a survey design that would cover the within‐habitat heterogeneity and give information about the occupancy–environment relationship that is general for the focal species and habitat type. It is justifiable to exclude the small resource units from the survey if they are seldom used by the species and if they therefore do not significantly influence its population dynamics (Loos et al., 2015; Zotz & Bader, 2011).

There were four aims in our study. The first aim was to test for differences in the future occupancies of (a) species with different landscape‐scale occupancy when using occupancy versus colonization–extinction models. The occupancy models are based on data from one point in time, while the colonization–extinction models are based on data from two points in time. As data suitable for occupancy models are available and frequently used for many species and many geographical areas, it is important to find out how the trends and magnitudes of change that occupancy models reveal differ from the ones revealed by colonization–extinction models for which data are currently scarce. Colonization–extinction models are expected to be more realistic for predicting changes as they focus on rate of changes (of occupancy). We hypothesize that the difference in the projected future occupancy between occupancy and colonization–extinction models is greater for a species with lower landscape‐scale occupancy because rare species can be expected to have slower colonization–extinction rates and therefore track changes in forest landscapes with a greater delay than common species. We further test for differences in projected future occupancies between modeling the data at (b) three different spatial modeling scales (cell, plot or patch) and (c) two resource‐unit resolutions (two different minimum diameters for deadwood to be included) to find out how scale and resolution influence predictions of future population trends. Inferences were made based on projections of occupancy of two model species in forest production land and in land set aside from production across the whole boreal zone of Sweden. The projections were obtained through stochastic simulations using the occupancy and colonization–extinction models fitted at different spatial scales and resource‐unit resolutions. Building the models was part of our fourth aim, specifically (d) to test which local and regional environmental variables explain the occupancy and colonization–extinction dynamics at different spatial scales and resource‐unit resolutions.

## 2 MATERIALS AND METHODS

### 2.1 Study patches and data collection

We obtained the large‐scale extensive data on colonization–extinction dynamics by surveying spruce deadwood and fruit bodies of the focal polypore fungi in 174 forest patches across southern and central Finland once in 2003–2005 (Nordén, Penttilä, Siitonen, Tomppo, & Ovaskainen, 2013) and then resurveying them in 2014. These two surveys revealed the colonization and extinction events that had taken place between the first and the second survey and constituted the data to estimate (parameters for) rate of change in occupancy in the colonization–extinction models. Data from the first survey formed the basis for the occupancy models.

**Figure 1:**
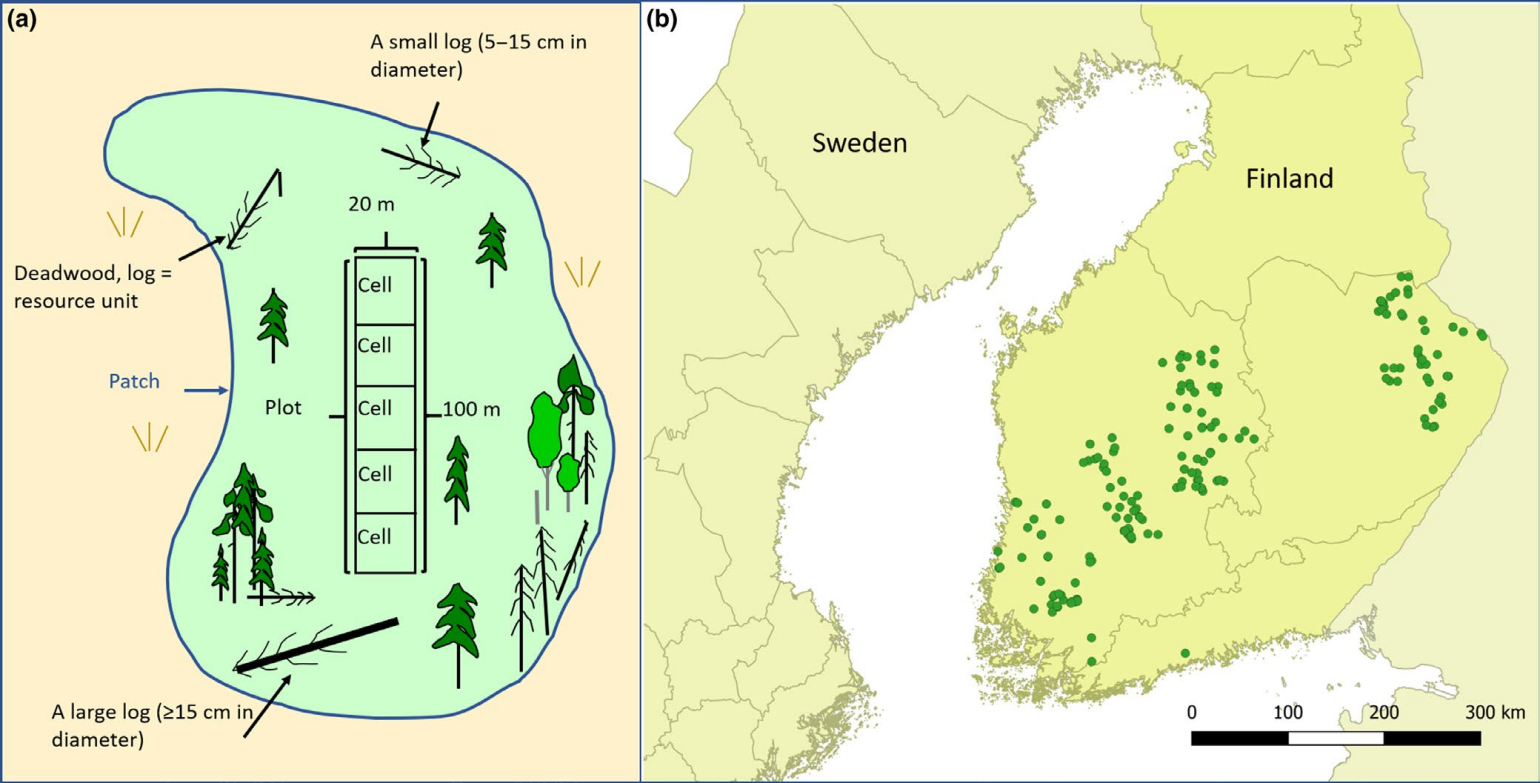
(a) The three spatial scales of data collection and modeling with a sample *plot* with five *cells* (20 × 20 m) in a forest *patch*. Small logs were only surveyed within the sample plot, while the large logs were surveyed across the whole patch. (b) The repeated survey data collected in 174 forest patches across boreal Finland used to build the occupancy and colonization–extinction models

A forest patch is a contiguous and homogeneous forest area that is surrounded by other land types or forests of different age or tree species (Figure 1). The survey plot was of the size 20 m × 100 m and subdivided into survey cells of 20 m × 20 m. All patches were dominated by Norway spruce (*Picea abies*) and covered a range of forest types: clear‐cuts with retention trees (53 patches, 16 of which had a plot, 16 × 5 cells), woodland key habitats (56 patches, 56 plots, 56 × 5 cells), and managed forests (65 patches, 65 plots, 65 × 5 cells). In each forest patch, in both surveys (2003–2005 and 2014), we surveyed the two fungal species (*Phellinus ferrugineofuscus* and *P. viticola*) and deadwood both in each cell and in the remaining patch area. See Appendix S1 for a detailed description of the data collection and the focal species.

### 2.2 Modeling occupancy and colonization–extinction

For each species, we fitted hierarchical Bayesian state‐space models to the presence–absence data of the species at three spatial modeling scales (cell, plot, and patch) and two deadwood resource resolutions (diameter ≥ 5 cm or ≥ 15 cm). We included covariates collected for different spatial modeling scales that we hypothesized would explain the occupancies and colonization–extinction dynamics of the focal species (Appendix S1). For the comparison with the colonization–extinction models, we also fitted occupancy models to data from the first survey.

A detailed description of the occupancy and colonization–extinction models at the cell level is provided in Appendix S1. The number of colonizations and extinctions recorded allowed including the effects of covariates on colonization probability of mature patches. For the extinction probability and the colonization probability of clear‐cut patches, only intercepts (i.e., the rate parameters) were estimated.

The covariates to retain in the final fitted models were determined with forward stepwise model selection. This model selection was based on overlap of 95% credible intervals with 0, reduction in deviance, and biological knowledge on the species, as suggested by Gelman and Hill (2007). The models were fit using OpenBUGS (Lunn, Spiegelhalter, Thomas, & Best, 2009) in R through the library R2OpenBUGS (Sturtz, Ligges, & Gelman, 2005). The data and computer code used for models, simulations, and statistical analyses are archived in the Swedish National Data Service, https://snd.gu.se/en.

### 2.3 Projecting polypore occupancies in the coming century

To answer our study questions, we utilized available projections of forest conditions on National Forest Inventory (NFI) plots in adjacent boreal Sweden between 2010 and 2,110, the nationwide Forestry Scenario Analysis by the Swedish Forest Agency (Claesson, Duvemo, Lundström, & Wikberg, 2015; Eriksson, Snäll, & Harrison, 2015; Appendix S1). Next, we projected the occupancy dynamics of the species for the same time period. For each polypore species, the final fitted occupancy model was utilized to initialize the occupancy states in the first time step, here 2010. We used 10‐year time steps to simulate the subsequent colonization and extinction dynamics on the NFI plots until 2,110 using the final fitted colonization–extinction model with its estimated parameter values. For investigating the effect of making projections based on occupancy models, the final fitted occupancy model was instead used for each time step.

All projections were made based on drawing 1,000 values from the joint posterior distribution of the parameters from the fitted models. All NFI plots with no dead spruce or those with ages 26–63 were given an occupancy probability of zero, because of the typical absence of spruce deadwood suitable for the species in forests of this age range (Mair et al., 2017).

## 3 RESULTS

### 3.1 Colonization and extinction events

We observed several colonization events, especially in the mature patches/plots/cells, and several extinction events, especially in the clear‐cut patches/plots/cells (Table 1). The clear‐cut forests lost almost all of their occurrences between the two surveys, and very few colonizations took place.

**Table 1:** Numbers recorded for each type of colonization–extinction history for different forest age classes across the varying spatial modeling scales and resource resolutions

Species	Age class	Spatial modeling scale	Resource resolution (diameter limit (cm)	Colonization–extinction history				Colonization rate	Extinction rate	Colonization rate/extinction rate	Occupancy
				11	10	00	01				
*Phellinus ferrugineofuscus*	Mature	Cell	5	0	5	357	17	0.05	1.00	0.05	0.04
			15	0	4	170	14	0.08	1.00	0.08	0.07
		Plot	5	1	4	79	12	0.13	0.80	0.16	0.14
			15	1	4	61	10	0.14	0.80	0.18	0.14
		Patch	15	4	7	66	17	0.20	0.64	0.32	0.22
	Clear‐cut	Cell	5	0	10	127	0	0.00	1.00	0.00	0.00
			15	0	4	38	0	0.00	1.00	0.00	0.00
		Plot	5	1	5	29	0	0.00	0.83	0.00	0.03
			15	0	5	15	0	0.00	1.00	0.00	0.00
		Patch	15	2	4	23	1	0.04	0.67	0.06	0.10
*Phellinus viticola*	Mature	Cell	5	1	10	338	30	0.08	0.91	0.09	0.08
			15	1	3	168	16	0.09	0.75	0.12	0.09
		Plot	5	3	6	69	18	0.21	0.67	0.31	0.22
			15	3	3	55	15	0.21	0.50	0.43	0.24
		Patch	15	7	6	61	20	0.25	0.46	0.53	0.29
	Clear‐cut	Cell	5	1	11	124	2	0.02	0.92	0.02	0.02
			15	1	3	38	0	0.00	0.75	0.00	0.02
		Plot	5	1	7	26	1	0.04	0.88	0.04	0.06
			15	1	6	12	1	0.08	0.86	0.09	0.10
		Patch	15	2	8	18	2	0.10	0.80	0.13	0.13

Note

A history of “’11” means that the patch was observed to be occupied at each survey event, whereas a history of “10” means that the patch was observed to be occupied at the first survey event but not the second. Rates are number of events observed divided by the number of events possible, and occupancy is the proportion of modeling units occupied in the second survey.

The species with the highest occupancy in the landscape, *P. viticola*, had higher colonization rates and higher ratios of colonization/extinction than the less frequent species, *P. ferrugineofuscus,* at all spatial modeling scales and both resource resolutions (Table 1). We observed the highest extinction rates for *P. viticola* at the fine resource resolution (≥5 cm) and the smallest spatial modeling scale (20 × 20 m). For *P. ferrugineofuscus,* the colonization and extinction rates were comparable between the fine resource resolution at the smallest spatial modeling scale and the coarse resource resolution at the largest spatial modeling scale. At the fine resource resolution, the colonization rate increased and extinction rate decreased with increasing spatial modeling scale. Similarly, at the coarse resource resolution, the largest spatial modeling scale had the highest colonization rates and the lowest extinction rates. Extinctions resulted both from host logs disappearing due to decomposition and stochastically where suitable logs were recorded in both surveys.

### 3.2 Summaries of fitted models

For *P. ferrugineofuscus*, the responses—probabilities of occurrence and colonization—were explained by the volume of spruce logs at the cell scale (here reflecting the presence of large logs), whereas the plot‐scale responses were explained by stand age and the patch‐scale responses by stand age or connectivity (Table S2‐1). The amount of data available allowed estimating the effects of one or two covariates (Table S2‐1), and hence, only one or two rounds of model selection were required. For *P. viticola,* the same responses were explained by the density of spruce logs (here reflecting many small logs) and connectivity at the cell and plot scales, and the density of spruce logs at the patch scale. The best‐fitting measure of connectivity for *P. ferrugineofuscus* was the presence/absence of old (≥120 years) spruce forests within a distance that corresponds to a mean dispersal distance of 1 km. For *P. viticola*, two measures of connectivity were important: the volume of spruce or presence/absence of spruce in old forests within a distance that corresponds to a mean dispersal distance of 10 km. Deadwood resource resolution had an influence on whether the density of logs was selected or not in the models for *P. viticola.*

### 3.3 Future projections

**Figure 2:**
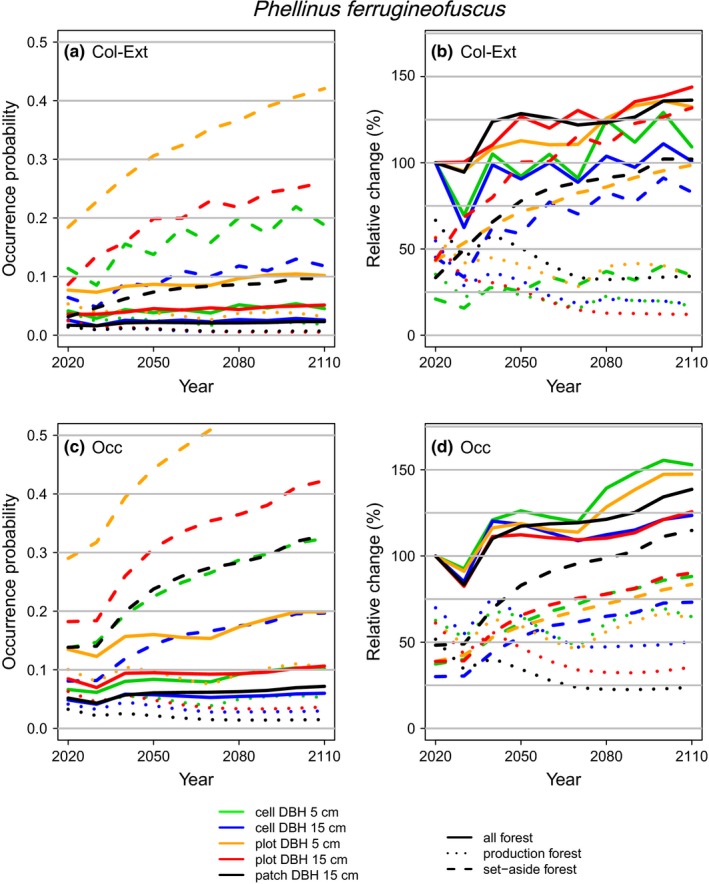
Projections of mean occurrence probability and relative change in occurrence for *Phellinus ferrugineofuscus* over the present century in response to forest management. Panels (a, b) are for projections based on the colonization–extinction models (*Col‐ext*) and panels (c, d) for those based on the occupancy models (*Occ*). The projections are based on averaging the results based on 1,000 simulations from the full posterior distributions of the fitted models

**Figure 3:**
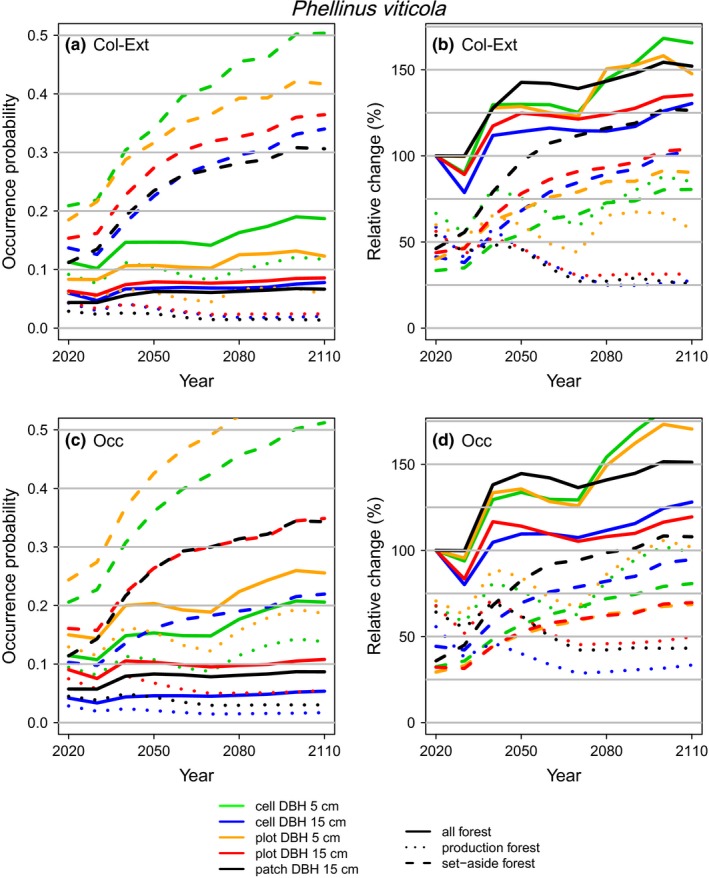
Projections of mean occurrence probability and relative change in occurrence for *Phellinus viticola* over the present century in response to forest management. Panels (a, b) are for projections based on the colonization–extinction models (*Col‐ext*) and panels (c, d) for those based on the occupancy models (*Occ*). The projections are based on averaging the results based on 1,000 simulations from the full posterior distributions of the fitted models

We predicted higher occurrence probabilities and relative changes from the projections based on the occupancy models than the colonization–extinction models. The general probability of our model species increasing across all forest land was higher using the occupancy models than the colonization–extinction models. The main reason for this was the predicted smaller decrease in production land when using the occupancy model (Figures 2 and 3, Table 2, and Figures S3‐1‐4). For both model species, the probability of an increase over all forest patches was unity for the projections based on the occupancy models but ranged from 0.59 to 0.96 for *P. ferrugineofuscus* and from 0.88 to 1 for *P. viticola* based on the colonization–extinction models. Occupancy models were more sensitive to the chosen resource resolution and spatial modeling scale than colonization–extinction models. Specifically, there was larger variation among the projection trajectories when using the occupancy models (Figures 2c,d and 3c,d) than when using the colonization–extinction models (Figures 2a,b and 3a,b).

**Table 2:** Mean change in occupancy across all forest land and in production land between 2020 and 2110 based on 1,000 simulations from the full posterior distributions of the fitted models

Species	Model type	Spatial modeling scale	Resource resolution (diameter in cm ≥ value)	Mean change in occupancy (95% Bayesian credible intervals; probability of increase), all forest land	Mean change in occupancy (95% Bayesian credible intervals; probability of decrease), production forest
*Phellinus ferrugineofuscus*	Colonization–extinction	Cell	5	0.003 (−0.010 to 0.012; 0.77)	−0.013 (−0.025 to −0.002; 1.00)
			15	0.000 (−0.010 to 0.008; 0.59)	−0.011 (−0.022 to −0.002; 1.00)
		Plot	5	0.025 (−0.018 to 0.049; 0.93)	−0.022 (−0.040 to −0.003; 0.99)
			15	0.016 (−0.004 to 0.026; 0.96)	−0.021 (−0.032 to −0.012; 1.00)
		Patch	15	0.006 (−0.007 to 0.011; 0.91)	−0.007 (−0.024 to −0.002; 1.00)
	Occupancy	Cell	5	0.035 (0.016 to 0.053; 1.00)	0.002 (−0.014 to 0.022; 0.47)
			15	0.012 (0.004 to 0.019; 1.00)	−0.012 (−0.023 to −0.002; 1.00)
		Plot	5	0.064 (0.045 to 0.080; 1.00)	0.005 (−0.017 to 0.029; 0.38)
			15	0.022 (0.014 to 0.026; 1.00)	−0.027 (−0.031 to −0.018; 1.00)
		Patch	15	0.020 (0.005 to 0.028; 1.00)	−0.017 (−0.025 to −0.009; 1.00)
*Phellinus viticola*	Colonization–extinction	Cell	5	0.073 (0.029 to 0.116; 1.00)	0.25 (−0.006 to 0.056; 0.08)
			15	0.017 (−0.010 to 0.043; 0.88)	−0.023 (−0.039 to −0.007; 1.00)
		Plot	5	0.039 (0.018 to 0.072; 1.00)	−0.003 (−0.020 to 0.026; 0.66)
			15	0.022 (0.007 to 0.036; 0.99)	−0.019 (−0.034 to −0.003; 0.99)
		Patch	15	0.022 (0.014 to 0.031; 1.00)	−0.015 (−0.026 to −0.005; 1.00)
	Occupancy	Cell	5	0.092 (0.070 to 0.107; 1.00)	0.044 (0.021 to 0.060; 0.00)
			15	0.012 (0.005 to 0.018; 1.00)	−0.011 (−0.023 to −0.001; 0.99)
		Plot	5	0.106 (0.091 to 0.114; 1.00)	0.057 (0.043 to 0.067; 0.00)
			15	0.018 (0.014 to 0.020; 1.00)	−0.020 (−0.028 to −0.012; 1.00)
		Patch	15	0.029 (0.020 to 0.035; 1.00)	−0.15 (−0.018 to −0.011; 1.00)

Note

Shown are also 95% Bayesian credible interval and probability of increase on all forest land and probability of decrease in production forest. All model types and resource resolutions predicted that there would be an increase in the set‐asides with a probability of 1.00.

For both model species, we projected an increase in the set‐asides across all spatial modeling scales and resource resolutions, owing to increasing density and volume of deadwood, stand age, and connectivity for set‐asides (Figure S4‐1,‐2). The relative change in occurrence across all the forest patches depended on the degree to which the positive trends in the set‐asides could compensate for the declines (where predicted) in the production land (Figures 2 and 3, Table 2, and Figures S3‐1‐4). We predicted higher occurrence probabilities for *P. viticola* than for *P. ferrugineofuscus*, and an increase in future occupancy was more likely for *P. viticola* than *P. ferrugineofuscus* across the two types of models, spatial modeling scales, and resource resolutions.

The differing forecasts of the occupancies of the two polypore species resulted from a combination of the general occupancies or colonization–extinction rates (Table 1) and the forecasts of the covariates of the fitted models (Table S2‐1). For a description of these links, see Appendix S1.

The magnitude of future increase in occupancy depended strongly on the spatial modeling scale (cell, plot, patch) (Figures 2 and 3). For both model species in mature forests, colonization rates were the lowest at the cell scale and increased going up via plot to patch scales, while the opposite was true for the extinction rates (Table 1). In clear‐cut forests, colonization events were rare and extinction events were common at all spatial modeling scales (Table 1). The consequence of these overall colonization and extinction rates were that the probability of future increase across all forest land was higher for both model species when the projections were conducted using the plot‐ or patch‐scale models than using the cell‐scale model (Table 2 and Figures S3‐1‐4). Applying the fine resource resolution (≥5 cm) colonization–extinction model for *P. viticola*, the probability of a decline in the production land was much lower using the cell‐ than the plot‐scale models (.08 vs. .66). However, here the .08 probability of decline also means a 1–.08 = .92 probability of increase, which is thus detected by the fine resource resolution.

Resource resolution (all or only large deadwood included) had a great impact on the future predictions. For *P. viticola*, we predicted clearly a more positive future population development with the fine resource resolution (≥5 cm) than with the coarse resolution (≥15 cm), and the precision of the prediction was higher for the coarse resolution (Table 2 and Figures S3‐3‐4). For *P. viticola,* we predicted, probably erroneously, a decline in the production land when we did not account for the small resolution deadwood units. For *P. ferrugineofuscus*, future decline in production land seemed certain based on the coarse resolution (both model types) but less probable (.47 and .38) based on occupancy models that used the fine resolution. Projections based on the occupancy models for *P. ferrugineofuscus* showed a decline in the production land only for the models based on the coarse resource resolution; for the fine resolution, the trends were more stable (Figure 2; Table 2, and Figure S3‐2). Projections based on the occupancy models for *P. viticola* similarly showed an almost certain (probability ≥ .99) decline in the production land only when we modeled at the coarse resource resolution; at the fine resolution, the probability of decline was zero (Figure 3; Table 2 and Figure S3‐4). We predicted the greatest increase in future occupancy when modeling at the fine resource resolution. The effect of resource resolution was less pronounced in the colonization–extinction models than in the occupancy models.

## 4 DISCUSSION

When making predictions for ecological systems, it is seldom clear from the outset which models to use and at what scale and resolution the modeling should be performed (Evans et al., 2013). Based on the joint posterior parameter distribution from hierarchical Bayesian models fitted to an extensive colonization–extinction dataset on deadwood‐dependent fungi, combined with realistic forest projection data, we show that the future trends predicted were sensitive to all four questions addressed—to the type of modeling performed, the landscape‐scale occupancy of the model species which affects their colonization–extinction rates, the spatial scale of model fitting, and the resolution of the resource‐unit data. For our model species, the resource‐unit resolution had a strong impact on the predictions especially for the species that frequently uses the smaller deadwood that was excluded from the coarse‐resolution data. Type of model (occupancy vs. colonization–extinction model) substantially affected the magnitude of the predicted change, while the effect of the spatial scale of model fitting was also considerable. We encourage the use of colonization–extinction models over occupancy models (or more generally, species distribution models, SDMs), modeling the process at the finest resource‐unit resolution that is utilizable by the species and conducting projections for the same spatial scale and resource resolution at which the model fitting is conducted.

### 4.1 Colonization–extinction models produce more realistic predictions

The occupancy models, corresponding to the frequently applied SDMs (Franklin & Miller, 2010), predicted what we believe is unrealistically positive population development. Our conclusion is based on knowledge about the study system and population development of the focal species during the recent decades. Compared to the colonization–extinction models, the occupancy models predicted higher occurrence probabilities and less steep future declines in the production land leading to more positive increases across all the forest land combined. Future declines are thus underestimated with occupancy models, especially if habitat amount is decreasing and the distances to dispersal sources are consequently increasing. Many of these species have slow life history which is often associated with rarity (Pilgrim, Crawley, & Dolphin, 2004). The occupancy models reflect the species distribution patterns which reflect the past rather than the current amount and connectivity of the habitat. Even more, occupancy SDMs often use data collected over a long time period during which the environment may change. The colonization–extinction models are more realistic because they reflect the rate of change from one time step to another. Their higher realism that they more mechanistically model the process leading to the occupancy pattern may also explain why they were less sensitive to the spatial scale modeled and the resource resolution. Limitations of their use may be the costs of making another survey of the system and the time span necessary for changes to take place.

The colonization–extinction rates observed at the patch and plot scales in this study were surprisingly high. Several local colonizations and extinctions had taken place during just 9–11 years, which challenges the view of very long time lags, from decades to much over 100 years, before a new equilibrium between the metapopulation and its environment is reached (Sverdrup‐Thygeson, Gustafsson, & Kouki, 2014). The high turnover rate may be partly explained by the ecology of our focal species which are not confined to very large or slowly decomposing dead trees. However, our results also suggest that in many species of deadwood‐dependent fungi, the delay in response to environmental change is shorter than previously thought. Despite this, metapopulation equilibrium cannot be assumed as the colonization–extinction models project lower future species occurrence than the occupancy models. This is especially so for *P. ferrugineofuscus* with a lower ratio of colonization/extinction. The species is thus tracking the changes in its environment with a delay, especially in the production forest with the highest rate of forest stand and deadwood turnover. The colonization–extinction models account explicitly for the temporal change, while occupancy models assume that the current occurrence pattern is at metapopulation equilibrium with the environment.

### 4.2 Considerations of appropriate spatial scale of model fitting

The predictions of the future population development depend strongly on the chosen spatial scale of the statistical model fitting. For the less frequent *P. ferrugineofuscus,* the predicted population increase by the year 2,110 ranged from 0% to 42%, depending on which of the three models were applied in the projections. We generally recommend conducting model fitting and simulation at a small spatial scale. This allows modeling and projecting the dynamics at the level at which the local population dynamics take place, including accounting for proximal variables within each patch and among patches. However, this recommendation of simulating detailed dynamics ignores the computational power required. Moreover, for making projections for a landscape or region, simulation of complete deadwood and population dynamics across the chosen spatial scale is required, ideally including dispersal between patches. However, for rare species with slow colonization–extinction dynamics and few occurrences on a small proportion of logs in each patch (here especially *P. ferrugineofuscus*), simulating detailed small‐scale deadwood dynamics may be inefficient. For such species, model fitting and projection simulation at a larger scale (here plot or patch) may be more appropriate, especially if the general question of the study concerns a landscape or region. Thus, conducting modeling and projection simulations at a more aggregated spatial resolution is acceptable. On the other hand, when modeling at a larger spatial scale, more distal predictors (e.g., stand age) are selected—these affect the species more indirectly than the proximal predictors they replace (Merow et al., 2014). The use of the more distal predictors may bring a higher level of uncertainty into the analyses, as it assumes a strong correlation between the distal predictors and the resources they replace. Moreover, if there is bias in this assumed correlation, then this bias is transferred into biased projections.

### 4.3 Appropriate resource resolution depends on the ecology of the study species

Resource‐unit resolution can have a considerable influence on the predictions of future population development. For *P. viticola,* the most striking difference in the projections was between using the fine‐ or coarse‐resolution deadwood data. Excluding the smaller deadwood units resulted in the conclusion that this species will decline in the production land, while when including them the decline was much reduced. For *P. ferrugineofuscus,* the population trends based on the coarse and fine deadwood data were more similar. This is because of the preference of *P. ferrugineofuscus* for larger‐diameter dead trees and consequently the models for this species having deadwood volume (influenced mostly by larger trees) as the significant covariate of resource availability. With different minimum sizes of deadwood inventoried, the deadwood quantities such as density and volume of deadwood—the measures of resource availability used as covariates in the models and projections—may also change (Hottola, Ovaskainen, & Hanski, 2009). However, it may also be wise to choose the resource‐unit resolution of analysis during the initial exploratory analysis. For example, a species may occur on a substrate of subordinate quality (e.g., small‐diameter logs) in a high‐quality area (old‐growth forest with high species abundance) resulting from mass effect. If erroneously assuming that it may occur on such substrate also in low‐quality areas (albeit at low probability) and if this substrate is very common in these lower‐quality areas, then one is likely to overestimate the future occupancy of this species, especially in low‐quality areas. This may be the case for *P. ferrugineofuscus* whose colonization probability increases with diameter (Jönsson, Edman & Jonsson, 2008), but which only occasionally occurs on the very common 5–10 cm logs. It may thus be justified to exclude the small resource units from the survey or analyses as their influence on population dynamics is minor (Loos et al., 2015). Another option, if data quantity allows, is to include the interaction between substrate size and forest age. See Appendix S1 for more discussion on resource resolution and species ecology.

### 4.4 Reliable prediction of future occupancy

Despite the differing occurrence probabilities and rate of change in future occupancies produced by the occupancy and colonization–extinction models, the direction of the change was usually the same. This is partly explained by the fact that the covariates selected for the colonization probabilities were, in most cases, the same as those selected for the occupancy probabilities. Arguably, precise predictions of biological responses to environmental change, especially if extrapolating beyond current conditions and into the future, require elaborate mechanistic process‐based models, driven by the detailed life history of the species (Evans et al., 2013). However, for essentially all species, including deadwood‐dependent fungi, the data required to parameterize such models are still lacking. Inaccurate estimation of the rate of change in occupancy will lead to severe bias in future projections, for example, when addressing the effects of global change (Dietrich et al., 2012) that may increase habitat turnover rates, making population persistence more dependent on a high number and good connectivity of habitat patches (Johst et al., 2011). The potential sources of bias in our predictions that we identified are detailed in Appendix S1. Nevertheless, with models for colonizations and extinctions accounting for key variables driving these metapopulation dynamics, such as the availability of suitable resource units, habitat quality (e.g., forest age), and spatial connectivity, we may detect the true future patterns and trends if they are strong.

## CONFLICT OF INTERESTS

There are no competing interests to report.

## AUTHORS' CONTRIBUTIONS

JN, PJH, JS, and TS conceived the ideas and designed methodology; JN, JS, and TS designed the data collection; PJH, LM, and JN analyzed the data; OK wrote the software to simulate deadwood decomposition; AL conducted the simulations of forest dynamics and management; and JN, PJH, and TS led the writing of the manuscript. All authors contributed critically to the drafts and gave final approval for publication.

## Data Availability

The data are archived in the Swedish National Data Service, https://snd.gu.se/en.
