# Supplementary material for: Occupancy versus colonization–extinction models for projecting population trends at different spatial scales
Source: Ecol Evol. 2020 Mar 3;10(6):3079–89. doi: 10.1002/ece3.6124 (PMC7083660; doi:10.1002/ece3.6124)
Supplement: Supplementary file 1 [file ECE3-10-3079-s001.docx]

**APPENDICES**

**APPENDIX S1.**

**Supplementary introduction**

THE STUDY SYSTEM: DEADWOOD-DEPENDENT FOREST FUNGI

Forest-living, deadwood-dependent decomposer fungi are an excellent system for testing the performance of different kinds of predictive models, including occupancy versus colonisation-extinction modelling, and the effects of spatial modelling scale and resource resolution on species projection performance. Wood-decomposer fungi are sessile species that may respond to environmental change with a delay (Gu et al. 2002, Paltto et al. 2006, Sverdrup-Thygeson et al. 2014). Snapshot occupancy data on wood-decomposer fungi are abundant, but in the current study we also utilize a large dataset on colonization-extinction dynamics collected over a wide ecological gradient. The resource units of the wood-decomposer fungi are dynamic in the sense that they have a lifespan of years to decades and even centuries (depending on tree species, tree size and environmental conditions). Wood-decomposer fungi must therefore balance extinctions from resource units with colonisations elsewhere to ensure metapopulation persistence (Gourbiere and Gourbiere 2002). In production forests, and especially for rare species, rates of local colonisation are expected to be lower and rates of local extinction higher than in natural-like forests, because production forests have much less deadwood (<10%) than natural-like forests (Jonsson et al. 2016) and the quality of the deadwood present usually does not meet the requirements of specialised species (Nordén et al. 2013). In more common species, the differences in colonisation and extinction rates between production and natural-like forests can be assumed to be smaller, and therefore the bias in projection when using occupancy models instead of colonization-extinction models can be assumed to be smaller. As deadwood rich set-aside forests are typically small in area and few in the landscape, many species of wood-decomposer fungi have small local populations and appear to be limited by dispersal (Edman et al. 2004, Norros et al. 2012). Furthermore, these fungi occur in hierarchically structured systems: they are restricted to resource units (dead trees) located in forest patches (Fig. 1 in the main text). Empirical data have usually been collected in sample plots of varying size, i.e. survey data that usually cover only a part of each forest patch. The fungi are to a varying extent specialised with respect to resource-unit characteristics, such as deadwood tree species, size, type and stage of decomposition (Nordén et al. 2013), and this gives an opportunity to test the effects of resource resolution on population projections.

**Supplementary material and methods**

DATA COLLECTION

In each forest patch, in both surveys (2003-2005 and 2014), we surveyed fungi and deadwood both in a survey plot and in the remaining patch area. The survey plot was of the size 20 m x 100 m and subdivided into survey cells of 20 m x 20 m (Fig. 1 in the main text). Within the cells we inventoried all deadwood with a diameter at breast height or base (depending on the deadwood type; referred to henceforth simply as diameter) ≥5 cm and length ≥1.3 m. For each spruce deadwood resource unit, we recorded the presence of fruit bodies of the focal species, spruce log diameter, length and decay stage. In the rest of the forest patch, i.e. in the remaining area outside the plot, we followed the same procedure but used a log diameter threshold of ≥15 cm. The lower resource-unit resolution in the remaining patch area was for covering the whole patch and for reaching a greater total number of patches than would have been possible if small-diameter deadwood was included everywhere.

All patches were dominated by Norway spruce (*Picea abies*) and covered a range of forest types: clear-cuts with retention trees (53 patches, 16 of which had a plot, 16 x 5 cells, Fig. 1 in the main text), age of dominant trees 5-20 years at the time of the second survey; brook-side and herb-rich woodland key habitats (56 patches, 56 plots, 56 x 5 cells), age 64-163 years, representing natural-like set-asides; and managed forests (65 patches, 65 plots, 65 x 5 cells) of varying age, 66-134 years, and intensity of management. Patch area ranged from 0.2 ha to 4.3 ha (mean 1.1 ha). We recorded the forest stand variables living spruce volume and stand age in the first survey. Stand age in the second survey was the number of years in between the two surveys added to the age in the first survey. Volume of living stand in 2014 was predicted based on the stand characteristics measured during the first inventory using the MOTTI forest stand simulator (Salminen et al. 2005, Hynynen et al. 2014). MOTTI is a stand-level forest management and decision support tool which includes tree-level models for predicting growth and mortality. Prediction models for stand dynamics and underlying data sets are presented in detail in Hynynen et al. (2014).

Focal polypore species

Our two focal polypore species have spruce deadwood as their main resource (Berglund et al. 2011), are very rarely found in patches 20-60 years old (because such forests lack suitable deadwood; H. Moor et al., unpublished data) which our empirical data did not cover; are absent on stumps and living trees (Berglund et al. 2011), and were expected to have differing occupancies and colonisation-extinction rates because of differing ecologies (see below). *Phellinus viticola* has fairly large and easily recognized perennial fruit bodies leading to high detectability (Halme and Kotiaho 2012). *Phellinus ferrugineofuscus* has annual-biennial fruit bodies but as they are large and also well recognizable long (up to years) after the fruit bodies have died, this species also has high detectability. Both species further usually produce many fruit bodies per log during many years so that the total fruiting period is several years, even >10 years (Ovaskainen et al. 2013). *Phellinus viticola* occurs on both small- and large-diameter logs (Juutilainen et al. 2011, Nordén et al. 2013). *Phellinus ferrugineofuscus* occurs more frequently on larger logs but occasionally also on the thinner logs (Nordén et al. 2013). *Phellinus ferrugineofuscus* is ecologically relatively specialised while *P. viticola* is rather generalised in its resource use. This likely explains the different proportions of occupied patches by the two species – *P. ferrugineofuscus* in 17% and *P. viticola* in 24% of the patches in 2014.

COVARIATES EXPLAINING OCCUPANCIES AND METAPOPULATION DYNAMICS

We hypothesised that covariates collected for different spatial modelling scales would explain the occupancies and colonization-extinction dynamics of the focal species. The resource unit covariates were density (ha^-1^) and volume (m^3^ ha^-1^) of spruce logs, their mean diameter and stage of decomposition. The patch-level covariates were living spruce volume, age of the living stand (range 4-206 years), wetness, slope and aspect, measured as in Mair et al. (2017). Landscape-scale covariates were connectivity to potential dispersal sources in the surrounding landscape, and annual temperature and precipitation (Mair et al. 2017). For our relatively rare and specialized focal species we hypothesised positive effects of all resource-, patch- and large-scale covariates on occupancy and colonization probability but negative effects on extinction probability. Two exceptions are stage of decomposition and wetness for which we expected a unimodal relationship. These hypotheses are supported by a large number of studies (e.g. Heilmann-Clausen and Boddy 2008, Abrego et al. 2017, Nordén et al. 2018). Connectivity was calculated using satellite-based estimates of spruce volume and forest age in the form of 25 m grid cell layers (Tomppo et al. 2008). We followed the procedure of Mair et al. (2017), weighing the volumes of spruce in surrounding grid cells up to 20 km from the focal patch by $\text{exp}\left( -\alpha d \right)$, where $d$ is the distance to the focal patch and alpha is the relevant spatial scale of connectivity. We tested two different thresholds in forest age for inclusion of grid cells in the connectivity calculation (grid cells with stand ages ≥100 years or ≥120 years). We also tested replacing the spruce volumes in the connectivity measure with the presence or absence of forest exceeding the age threshold in each grid cell. As there is limited information available on species’ dispersal distances we further tested three different values for $\alpha$ (0.1, 0.2 and 1), representing a mean dispersal distance of 10, 5 and 1 km, respectively. All covariates were log transformed, centred and standardised to have a mean of zero and a variance of unity.

Estimating the detection probability

One potential pitfall in models based on survey data is failing to account for unobserved species presences which can result in biased estimates of population trends and extinction probabilities (Kéry et al. 2006, Rhodes et al. 2006). Non-detection has likely a minimal effect on our projections as we accounted for it in several ways. Imperfect detection because of species characteristics, surveyor behaviour or sampling design can result in incorrect conclusions about population trends if not accounted for with the sampling and model design (MacKenzie et al. 2003, Altwegg et al. 2008). Firstly, in fungi, the species might not be fruiting at the time of the survey even if it is present as mycelia inside the dead tree. However, our focal species have similar mycelial and fruit-body prevalences as well as short time delays from mycelial colonisation to fruiting (Ovaskainen et al. 2013) which makes fruit body data meaningful and reliable. Secondly, the species might go non-detected even if it is present and fruiting, but we accounted for this in our models. Finally, a subset of the resource units may be included in the survey (e.g. based on minimum diameter), which we show can result in an opposite conclusion as compared with including all resource units: *P. viticola* was predicted to decrease in production forests when its smallest resource units were excluded, but predicted to increase when all the resource units were included.

A further hierarchical level in Bayesian modelling allows the unobserved “true” state of the population to be linked to the observed state via a model for the observation process and thus accounting for detectability (Harrison et al. 2011). For estimating the detection probability of the fruit bodies of the two polypore species, a larger survey plot of 4 ha (64 cells of the size 25 m x 25 m) in an old-growth forest patch in southern Finland was first surveyed in 2002 and partly (15 cells) resurveyed twice in 2014. The principle for detectability data collection is similar as in the general survey, and hence the detectability estimate obtained was used in the modelling, see below. In 2014, a field team of two experts first carried out a regular resurvey without knowledge about the subsequent second resurvey. In the second resurvey, the ‘intensive resurvey’, the task was to do as thorough a survey as possible, taking the time needed, in a team of four experts that now knew the purpose of the intensive resurvey was to collect data for estimating detectability. We fit detectability models to these data assuming the occupancies recorded during the intensive resurvey represented the true occupancies of the polypore species encountered during the surveys. As the density of deadwood is considerably larger overall in this old-growth forest than in a typical managed forest, we included deadwood density as a covariate in the detectability models. The mean detection probability across all polypore species recorded in the intensive resurvey was estimated to be 0.9 for the mean deadwood density in managed forests, and >0.9 in forests with a low density of dead wood. In the colonisation-extinction and occupancy models, we therefore used a patch-specific detection probability 0.9.

modelLING OCCUPANCY AND Colonisation-extinction

For each species, we fitted hierarchical Bayesian state-space models to the presence-absence data of the species at three spatial modelling scales (cell, plot and patch) and two deadwood resource resolutions (diameter ≥5 cm or ≥15 cm). We included covariates collected for different spatial modelling scales that we hypothesised would explain the occupancies and colonization-extinction dynamics of the focal species. For the comparison with the colonisation-extinction models we also fitted occupancy models. We used data from the second survey (174 patches surveyed both in 2003-2005 and 2014), and, for obtaining more reliable estimates of occupancy (but not colonization-extinction requiring two surveys) on clear-cuts, especially in relation to clear-cut age, we further included data from 19 patches from the first survey (2003-2005) that were not re-surveyed in 2014, and 22 patches that were cut between the two surveys and therefore only surveyed in the first survey.

In the modelling of species occupancy in clear-cuts, we only tested the effects of stand age and connectivity, and used all first-survey and resurvey data from clear-cut patches. We hypothesized (i) a negative age effect with time since cutting, and (ii) a positive effect of connectivity to surrounding dispersal sources. We did not test for the effect of deadwood quantity in clear-cut patches as most of the dead wood in these patches is made up of logging residues which, based on our data, are very seldom used by the focal species.

The response variables in the models (probabilities of occurrence, colonisation and extinction) were adjusted to a time scale of 10 years (only colonisation and extinction concerning change through time) and a spatial modelling scale of one hectare (Appendix S1). This was conducted by offsetting for the differing numbers of years between the surveys and the varying survey areas (cell, plot or patch). In the cell-level models, we included a random effect for the patches, which was assumed to be normally distributed with mean zero and a variance that was estimated during model fitting. In the projections we assumed a colonisation probability of zero and extinction probability of unity at such locations.

We fitted separate models at each of the cell-, plot- and patch scales and we detail the cell-scale model. The plot- and patch-scale models are simplifications of this model with the finest level indexing instead at the plot and patch scale, respectively.

For the cell-scale models we define $Z_{i,j,t}$ as the occupancy state of the focal species in cell $i$ in patch $j$ during survey period $t$. We assume that $Z_{i,j,t} \sim\mathrm{Bernoulli}\left( \psi_{i,j,t} \right)$. For the first survey period $\psi_{i,j,t}=\omega$ and in the following period:

$\psi_{i,j,t}=\left( 1-Z_{i,j,t-1} \right)c_{i,j,t}^{*}+Z_{i,j,t-1}\left( 1-e_{i,j,t}^{*} \right)$,

where $c_{i,j,t}^{*}$ and $e_{i,j,t}^{*}$ give the colonisation and extinction probabilities, respectively. They have been offset to correct for the different numbers of years between the surveys and the different cell areas (20 m x 20 m or 25 m x 25 m), such that:

$$c_{i,j,t}^{*}=1-\left( 1-c_{i,j,t} \right)^{n_{i,j,t}a_{i,j,t}}$$

$$e_{i,j,t}^{*}=1-\left( 1-e_{i,j,t} \right)^{\frac{n_{i,j,t}}{a_{i,j,t}}}$$

where $n_{i,j,t}$ gives the number of years between the surveys divided by 10 (i.e. scaled by the typical number of years) and $a_{i,j,t}$ gives the cell area divided by 400 (i.e. scaled by the typical cell size in m^2^). For a cell with ten years between the two surveys and an area of 400 m^2^, $c_{i,j,t}^{*}=c_{i,j,t}$ and $e_{i,j,t}^{*}=e_{i,j,t}$. If the forest in the cell had not been clear-cut we assumed that:

$$\mathrm{cloglog}\left( c_{i,j,t} \right)=\delta_{2}+\sum_{k} \beta_{k}^{(C)}X_{k,i,j,t}^{(C)}+\sum_{l} \beta_{l}^{(P)}X_{l,j,t}^{(P)}+\gamma_{j,t}$$

and that $\mathrm{cloglog}\left( e_{i,j,t} \right)=\varepsilon_{2}$. We chose to use the complementary log-log link function, $\text{cloglog}$, as due to its asymmetrical nature it is better suited than the more conventional logistic link function to cases where the probabilities are very large or very small (Golding et al., in preparation). Due to data limitations we could not include covariates or random effects in the models for the extinction probabilities or the colonisation probability for cells in forest patches that had been clear-cut (either before the first survey or between the two survey events) and intercept only models were used in these cases. Specifically, we estimated separate parameters for clear cuts, $\text{cloglog}\left( c_{i,j,t} \right)=\delta_{1}$ and $\mathrm{cloglog}\left( e_{i,j,t} \right)=\varepsilon_{1}$. The $k$ cell-scale covariates used in the model for the colonisation probability are given by $X_{k,i,j,t}^{(C)}$ with corresponding parameter values $\beta_{k}^{(C)}$. The $l$ patch-scale covariates are given by $X_{l,i,j,t}^{(P)}$ with corresponding parameter values $\beta_{k}^{(P)}$. We also include a patch-scale random $\gamma_{j,t}$which was assumed to be normally distributed with mean zero and variance $\sigma^{2}$. In cases where colonisation and extinction probabilities were observed to be 0 and 1, respectively (see Table 1), we used these probabilities in the simulations.

Finally, we define $Y_{i,j,t}$ as the observed occupancy state of cell $i$ in patch $j$ during survey period $t$. For the observation model we assume that $Y_{i,j,t} \sim\mathrm{Bernoulli}\left( Z_{i,j,t}p \right)$ where $p$ gives the detection probability. This detection probability was estimated based on an intensive control study (see *Estimating the detection probability* above) and a value of 0.9 was used.

For the cell-scale models it was necessary, due to statistical non-independence, to include a random patch effect term. At the higher spatial modelling scales (plot and patch) it was not possible to include such random effects. This random effect term has the advantage that it will capture between patch variation that is not explained by the covariates included in the models, for instance due to unmeasured covariates, habitat differences at the time of establishment but not necessarily at the time of the inventories, or competitive exclusion by species already established on some patches (Berglund et al. 2009b). True absences on some suitable patches can also be a consequence of stochasticity in the dynamics of either the species (Snäll et al. 2005) or their host trees (Jonsson 2000) or both. Not accounting for such variability has been shown to bias model-based predictions (Berglund et al. 2009a), especially for resource-unit restricted species such as wood-decomposer fungi.

PROJECTing FOREST CONDITIONS the coming century

We made projections of the distribution of the resource of our focal species on 17,383 National Forest Inventory (NFI) plots spread across the boreal zone of Sweden between 2010 and 2110 under a ‘business as usual’ scenario utilizing the recent nationwide Forestry Scenario Analysis by the Swedish Forest Agency (Claesson et al. 2015, Eriksson et al. 2015). In this scenario, 16% of forest land is protected in state reserves, patches voluntarily protected by individual landowners or areas set aside from forestry, retention patches. We refer to the collection of these non-production patches as set-asides in what follows. The set-asides are situated in forest landscapes otherwise dominated by clear-cutting forestry. The NFI plots are circular and have a diameter of 7 or 10 m but for all the projections we first scaled all variables to values at one hectare.

Projections of forest management and dynamics were made using the multiple-use forestry planning tool Heureka (Wikström et al. 2011). For the projections the RegWise software (version 2.2) was used. RegWise is a software component within the Heureka suite of forest decision support tools (Wikström et al. 2011). The core of Heureka is made up of empirical individual models for tree growth (see Fahlvik et al. 2014), ingrowth (Wikberg 2004) and mortality (Elfving 2014) together simulating tree layer development in five-year time steps. In RegWise forest management actions are steered by a rule-based simulation framework. The harvest level – and consequently the development of the forest – is controlled by specified management programmes (silviculture and harvest activities) and the actual growth in each time period. Different management programmes are specified for, e.g., different forest owner categories, tree species mixtures and site factors. Tree retention practices at final felling were also included. The forest projection output data included stand age, a covariate used in the modelling, living spruce volume which was utilised in our connectivity calculations and spruce deadwood production generated by natural tree mortality. We initialized the projections with the amounts and properties of spruce deadwood observed on the NFI plots during 2008-2012. We simulated decomposition of the deadwood based on the one-time chronosequence method described in Harmon et al. (2000). When deadwood enters the fourth stage of decomposition (based on the Swedish decay stage classification (Sandström et al. 2007), it is removed from the simulations as our focal species do not produce fruit bodies on such highly decomposed wood. We do not differentiate between standing (snags) and downed (logs) deadwood in the simulations but used the proportion of spruce logs (out of all dead spruce) in the NFI data to determine how much to multiply the deadwood quantities by in the covariates for our focal species. These multipliers were estimated as 0.59 for the volume of spruce dead wood and 0.64 for the density of dead spruce trees. Forest connectivity projections were done as in Mair et al. (2017).

All the model parameters were given priors with a mean of zero and a standard deviation of 0.01, except for sigma which was given a uniform prior between zero and five. In all cases three MCMC chains were run for 3000 iterations with a burn-in of 1000 iterations and a thinning of 100. Convergence was assessed visually based on the results of the three chains. In all cases convergence was reached after 3000 iterations.

projectiNG polypore occupancies the coming century

We projected the occupancy dynamics of our focal species for 2010-2110. For each polypore species the final fitted occupancy model was utilised to initialise the occupancy states in the first time step, here 2010. As the average time between our surveys was 10 years, we used 10-year time steps to simulate the subsequent colonisation and extinction dynamics in the NFI plots until 2110 using the final fitted colonisation-extinction model with its estimated parameter values. Offsets were used on the occupancy and colonisation and extinction probabilities to account for the different sizes of the NFI plots (7 and 10 m radii). Finally, area factor scaling, accounting for different densities of NFI tracts across Sweden, was used to compute means and totals of these hectare-scale values. Although our model fitting, projections and decomposition modelling are in terms of deadwood with diameter ≥5 cm at the finest resolution, the NFI data used for initialising the projections are in terms of deadwood with diameter ≥10 cm. Visual inspection of the modelling output showed that after ten years the effect of the missing small pieces (<10 cm) on the deadwood projections was minimal and we thus show the results between 2020 and 2110.

**Supplementary results**

FUTURE PROJECTIONS

The differing forecasts of the occupancies of the two polypore species resulted from a combination of the general occupancies or colonization-extinction rates (Table 1 in the main text) and the forecasts of the covariates of the fitted models (Table S2-1). Projections for the density of logs showed an increase in the production land when based on the fine resolution deadwood data, but a decrease when based on the coarse resolution data. However, the deadwood volume was predicted to decline in the production land at both resolutions (Fig. S4-1). This was because of a declining stand age in the production land over the coming decades, with the steepest decline between 2020 and 2050 (Fig. S4-2). The clear-cutting of old (>120 years) unprotected forests resulted in fewer large logs that would contribute to the deadwood volumes, whereas the density of logs in the production land would still increase owing to the self-thinning of younger forests. This abrupt initial decline in the ages in the production land had a strong influence on the connectivity measures which affected both the production land and the set-asides. After 2050 the connectivity was predicted to increase again, returning or exceeding the initial levels.

**Supplementary discussion**

APPROPRIATE RESOURCE RESOLUTION DEPENDS ON THE ECOLOGY OF THE STUDY SPECIES

Local dynamics of wood-decomposer species are influenced by the characteristics and abundance of the resource units the species primarily uses. Of the cell-scale covariates selected in our fitted models for the colonisation probability, *P. viticola* responded to the density of logs, whereas *P. ferrugineofuscus* responded to the volume. At this smallest spatial modelling scale (20 m x 20 m cells) a large volume at one cell, relative to that at other cells, will often be caused by one or two large logs. Higher log densities, on the other hand, will often be made up of several smaller deadwood units. In a previous study of colonisation and extinction events at the individual log level, Jönsson et al. (2008) found that for *P. viticola,* colonisation probability increased with connectivity to occupied logs regardless of log size, whereas for *P. ferrugineofuscus,* colonisation probability increased with log diameter. During the first round of model selection, we found a positive effect of mean log diameter for *P. ferrugineofuscus* when based on the cell-level fine scale deadwood data (results not shown), but once volume had been included in the model this effect was no longer significant. At the level of individual dead trees, Jönsson et al. (2008) found that *P. viticola* had a substantially lower log-level extinction risk than *P. ferrugineofuscus* (with annual mortality rates of 13.1% and 29.6%, respectively) on similar-sized logs, probably reflecting the longer fruiting period and later peak in fruiting in *P. viticola* than *P. ferrugineofuscus* (Jönsson et al. 2008, Nordén et al. 2013, Ovaskainen et al. 2013). In our data, *P. viticola* had higher extinction rates than *P. ferrugineofuscus* at the cell, plot and patch scale especially when including also 5-15 cm logs. The difference is likely because in our data *P. viticola* occurs on average on smaller, fast-decomposing logs than P*. ferrugineofuscus* that leads to more frequent extinctions in *P. viticola*. The rate of deterministic extinction due to log disappearance increases with decreasing log size.

POTENTIAL BIAS IN MODEL PREDICTIONS

Our predictions of the future population trends may be biased for several reasons. Firstly, connectivity decreases for 20 years after 2020 (Fig. S4-2). Some populations acting as dispersal sources will disappear during that time which will slow down the colonization rates more than what is expected by our models. Secondly, the forest currently voluntarily set aside may in fact not be set aside until the year 2100. For example, the mean length of private landownership is 22.5 years (Ingemarson et al. 2006) and new owners often start by cutting the oldest forest (Eriksson 2008). Thirdly, we ignored edge effects from clear cuts into set-asides which will have a negative effect on sensitive wood-decomposer fungi (Ruete et al. 2017). Fourthly, we may have misestimated the colonisation and occurrence probabilities in deadwood-rich forests as our models are based on field data from forests with low or intermediate amounts of deadwood. Fifthly, we assumed that *P. ferrugineofuscus* colonizes all dead trees with diameter ≥5 cm with the same probability while other studies have shown that the colonization and occurrence probabilities increase with log diameter for this species (Jönsson et al. 2008, Nordén et al. 2013). Finally, future climate is likely to be adverse for our model species (Mair et al. 2018) even if our data lacked the power to detect climate effects.

**APPENDIX S2**

**Table S2-1.** Covariates and associated parameter estimates in the final fitted models. For the colonisation-extinction models, the covariates are for the colonisation probability only. Covariates enclosed in square brackets were those selected for the occupancy models on cut patches. All covariates were standardised to have a mean of zero and standard deviation of one.

| Species | Model type | Spatial modelling scale | Resource resolution  (diameter in cm ≥ value) | | Covariates and associated parameter estimates (95% Bayesian credible interval) |
| --- | --- | --- | --- | --- | --- |
| *P. ferrugineofuscus* | colonisation-extinction | cell | | 5 | deadwood volume 1.32 (0.47-2.54) |
|  |  |  | | 15 | deadwood volume 0.68 (-0.18-1.82) |
|  |  | plot | | 5 | stand age 0.46 (-0.19-1.21) |
|  |  |  | | 15 | stand age 1.25 (0.12-3.42) |
|  |  | patch | | 15 | connectivity^*^ 0.45 (-0.06-1.05) |
|  | occupancy | cell | | 5 | deadwood volume 1.23 (0.44-2.32) |
|  |  |  | | 15 | deadwood volume 0.66 (-0.22-1.82) |
|  |  | plot | | 5 | stand age 0.51 (-0.10-1.18) |
|  |  |  | | 15 | stand age 1.02 (0.17-2.02) |
|  |  | patch | | 15 | stand age 0.57 (0.06-1.14) |
| *P. viticola* | colonisation-extinction | cell | | 5 | density of dead trees 1.20 (0.48-2.20) and connectivity^†^ 0.99 (0.13-2.19) |
|  |  |  | | 15 | connectivity^‡^ 2.91 (-0.02-1.01) |
|  |  | plot | | 5 | density of dead trees 0.47 (0.75-6.58) |
|  |  |  | | 15 | connectivity^‡^ 0.58 (-0.11-1.48) |
|  |  | patch | | 15 | density of dead trees 1.14 (0.46-2.03) |
|  | occupancy | cell | | 5 | density of dead trees 1.08 (0.49-1.79) and connectivity^†^ 0.77 (0.04-1.63) [connectivity^†^ 1.31 (0.50-2.35)] |
|  |  |  | | 15 | connectivity^‡^ 2.02 (0.47-4.58) [connectivity^‡^ 2.05 (-0.98-6.40)] |
|  |  | plot | | 5 | density of dead trees 0.54 (0.08-1.03) [connectivity^†^ 0.71 (0.16-1.30)] |
|  |  |  | | 15 | connectivity^‡^ 0.56 (-0.05-1.28) |
|  |  | patch | | 15 | density of dead trees 0.90 (0.45-1.38) |

^*^ connectivity based on presence/absence of spruce forests (according to a GIS-based layer for spruce volume (Tomppo et al. 2008)) ≥120 years (according to a GIS-based layer for forest age (Tomppo et al. 2008) and an α value of 0.1

^†^ connectivity based on volume of spruce (according to a GIS-based layer for spruce volume (Tomppo et al. 2008) in forests ≥120 years (see ^*^ above) and an α value of 1

^‡^ connectivity based on presence/absence of spruce forests (see ^*^ above) ≥120 years (see ^*^ above) and an α value of 1

**APPENDIX S3**


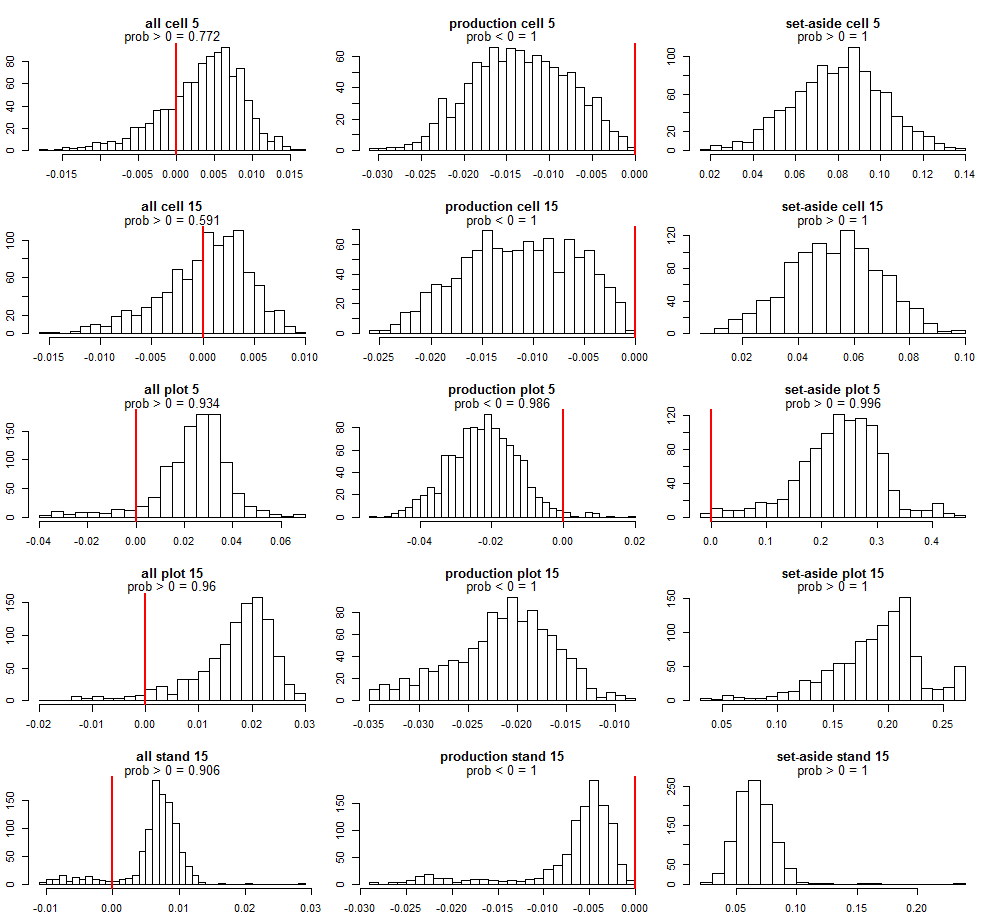


**all patch 15** prob > 0 = 0.906

**prob**

**production patch 15** prob < 0 = 1

**prob**

**set-aside patch 15** prob > 0 = 1

**prob**

**Fig. S3-1.** Probability of an increase over all patches (left), a decrease in the production land (middle) and an increase in the set-asides (right) based on the colonization-extinction models for *Phellinus ferrugineofuscus*.


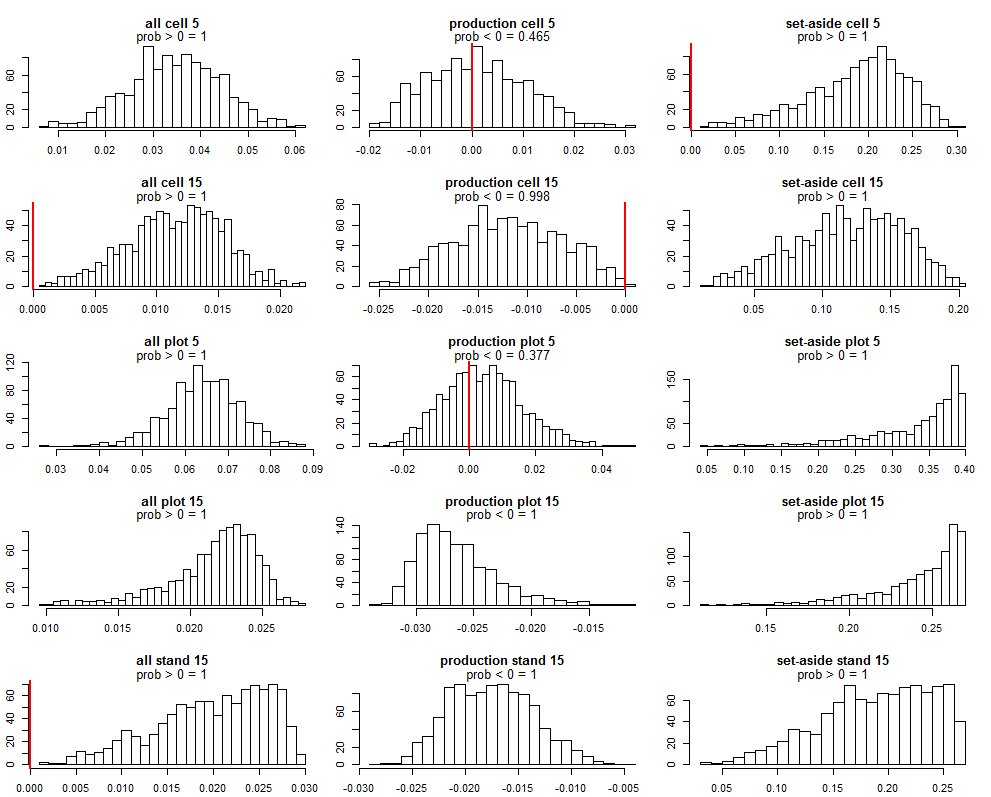


**all patch 15** prob > 0 = 1

**prob**

**production patch 15** prob < 0 = 1

**prob**

**set-aside patch 15** prob > 0 = 1

**prob**

**Fig. S3-2.** Probability of an increase over all patches (left), a decrease in the production land (middle) and an increase in the set-asides (right) based on the occupancy models for *Phellinus ferrugineofuscus*.


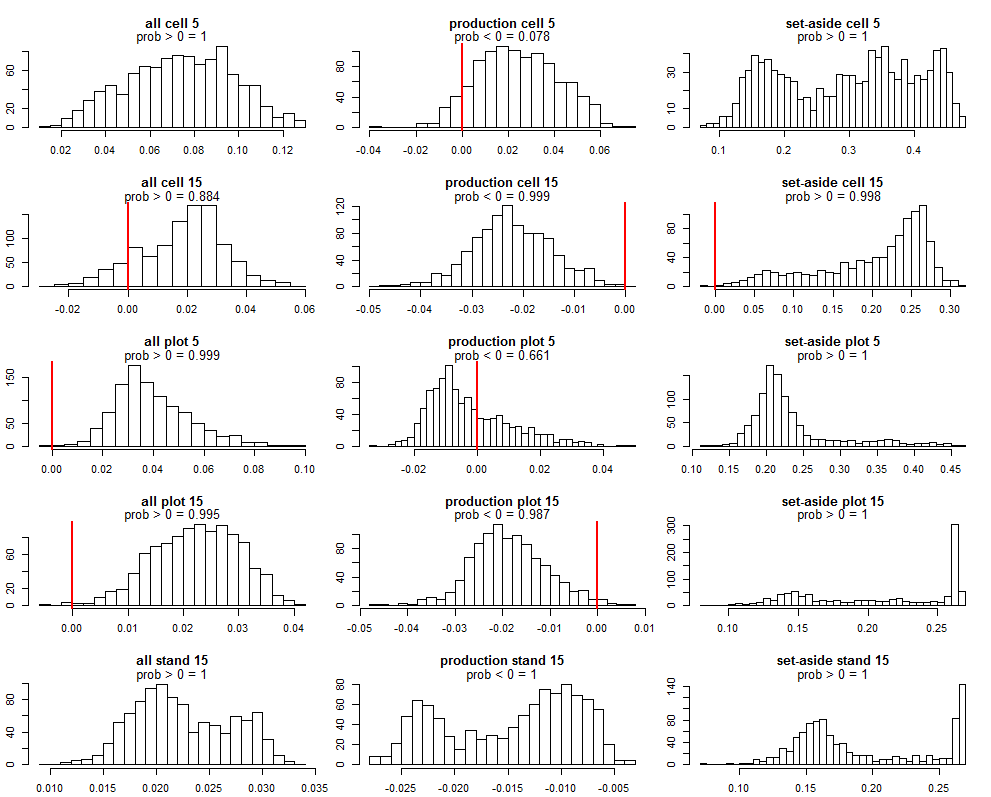


**all patch 15** prob > 0 = 1

**prob**

**prob**

**set-aside patch 15** prob > 0 = 1

**prob**

**production patch 15** prob < 0 = 1

**prob**

**Fig. S3-3.** Probability of an increase over all patches (left), a decrease in the production land (middle) and an increase in the set-asides (right) based on the colonization-extinction models for *Phellinus viticola*.


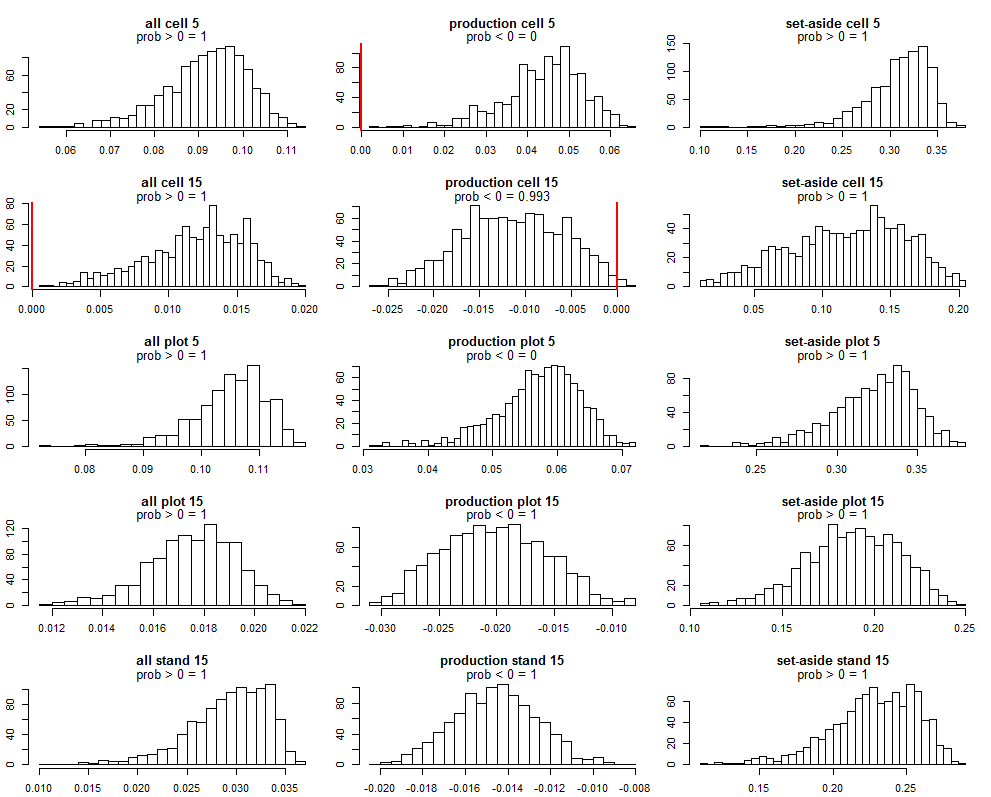


**all patch 15** prob > 0 = 1

**prob**

**set-aside patch 15** prob > 0 = 1

**prob**

**production patch 15** prob < 0 = 1

**prob**

**Fig. S3-4.** Probability of an increase over all patches (left), a decrease in the production land (middle) and an increase in the set-asides (right) based on the occupancy models for *Phellinus viticola*.

**APPENDIX S4**

**
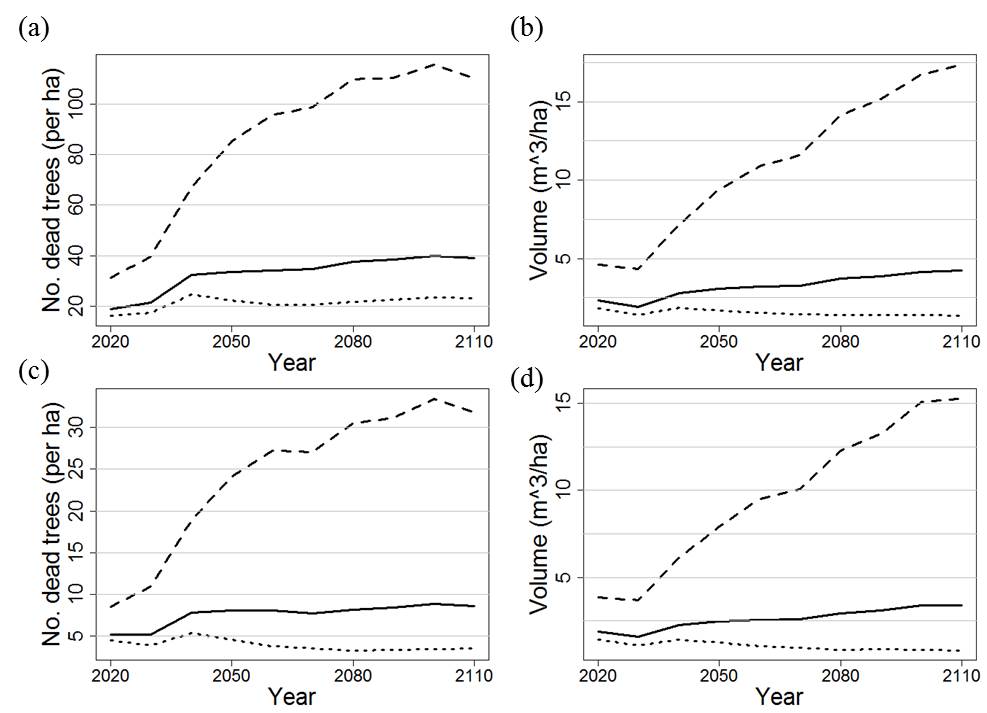
**

**Fig. S4-1.** Projections of the spruce deadwood covariates driving the species projections using the colonisation-extinction and occupancy models (see Table S2-1). Panels a and b give respectively the density and volume of logs with a diameter ≥5 cm in all forest land (solid lines); production forests (dotted lines); and set-asides (dashed lines). Panels c and d give the corresponding values for the coarse resolution deadwood data (diameter ≥15 cm). Note that when deadwood reaches the fourth stage of decomposition (based on the Swedish decay stage classification (Sandström et al. 2007), it is excluded. We do not differentiate between standing (snags) and downed (logs) deadwood in the simulations but used the proportion of spruce logs (out of all dead spruce).


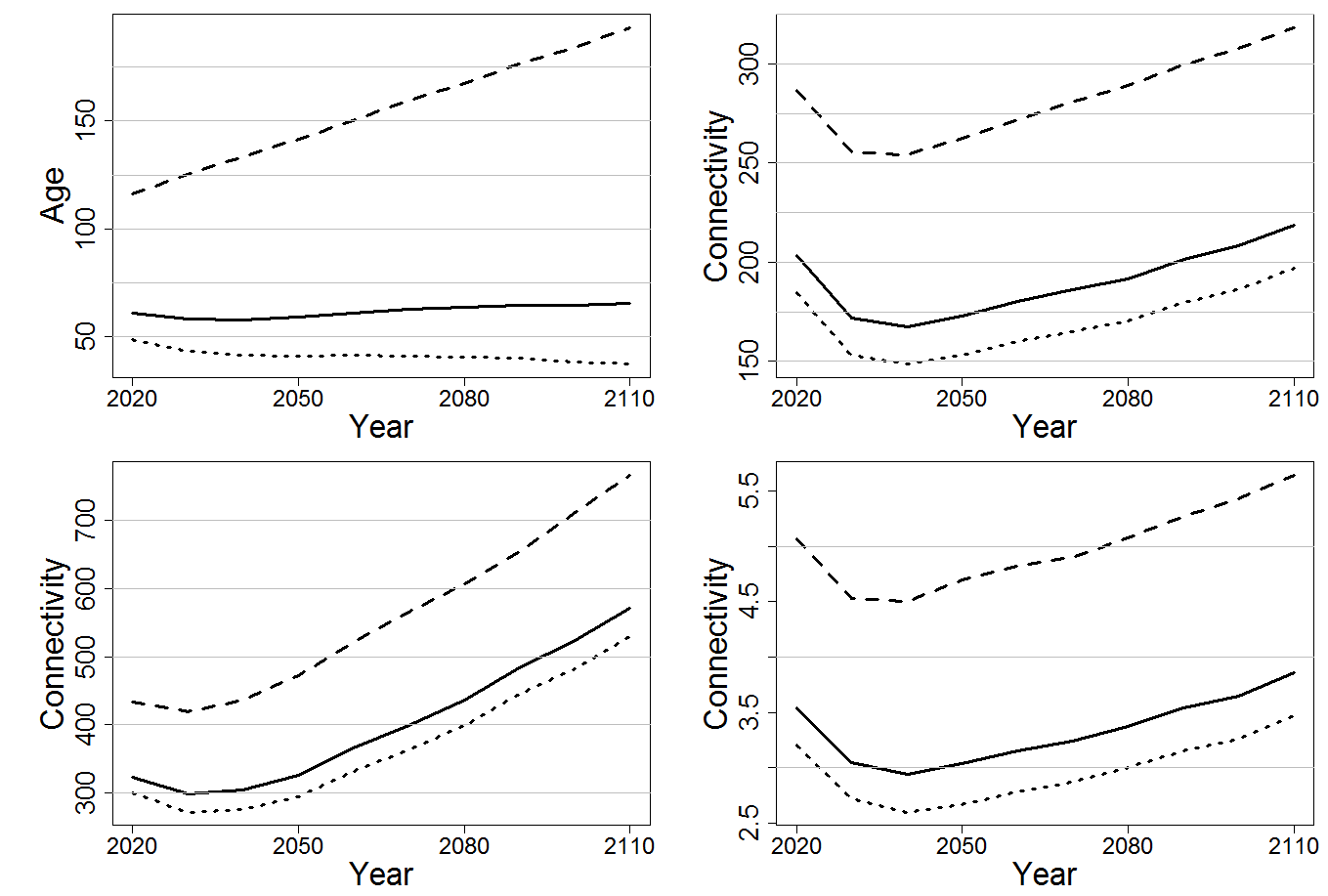


**Fig. S4-2.** Projections of stand age and connectivity covariates driving the species projections using the fitted colonisation-extinction and occupancy models (see Table S2-1). Panel a gives the stand age in all forest land (solid lines); production forests (dotted lines); and set-asides (dashed lines). Panel b-d give the connectivity measures used in the final models: (b) connectivity based on presence/absence of spruce forests ≥120 years and an α value of 0.1; (c) connectivity based on volume of spruce in forests ≥120 years and an α value of 1; and (d) connectivity based on presence/absence of spruce forests ≥120 years and an α value of 1.

**References**

Abrego, N., M. Christensen, C. Bässler, A. Ainsworth, and J. Heilmann-Clausen. 2017. Understanding the distribution of wood-inhabiting fungi in European beech reserves from species-specific habitat models. Fungal Ecology **27**:168-174.

Altwegg, R., M. Wheeler, and B. Erni. 2008. Climate and the range dynamics of species with imperfect detection. Biology Letters **4**:581-584.

Berglund, H., J. Hottola, R. Penttilä, and J. Siitonen. 2011. Linking substrate and habitat requirements of wood-inhabiting fungi to their regional extinction vulnerability. Ecography **34**:864-875.

Berglund, H., and B. G. Jonsson. 2008. Assessing the extinction vulnerability of wood-inhabiting fungal species in fragmented northern Swedish boreal forests. Biological Conservation **141**:3029-3039.

Berglund, H., R. B. O'Hara, and B. G. Jonsson. 2009a. Quantifying Habitat Requirements of Tree-Living Species in Fragmented Boreal Forests with Bayesian Methods. Conservation Biology **23**:1127-1137.

Berglund, H., B. O’Hara, and B. G. Jonsson. 2009b. Quantifying Habitat Requirements of Tree-Living Species in Fragmented Boreal Forests with Bayesian Methods. Conservation Biology.

Claesson, S., K. Duvemo, A. Lundström, and P. E. Wikberg. 2015. Forest impact analysis 2015 - SKA 15 (Skogliga konsekvensanalyser - SKA 2015). . Swedish Forest Agency.

Edman, M., M. Gustafsson, J. Stenlid, B. G. Jonsson, and L. Ericson. 2004. Spore Deposition of Wood-Decaying Fungi: Importance of Landscape Composition. Ecography **27**:103-111.

Elfving, B. 2014. Modelling of natural mortality in Heureka (Modellering av naturlig avgång i Heureka). Swedish University of Agricultural Sciences.

Eriksson, A., T. Snäll, and P. J. Harrison. 2015. Analys av miljöförhållanden - SKA 15., Swedish Forest Agency.

Eriksson, L. 2008. Treatment decisions in privately owned forestry. Swedish University of Agricultural Sciences, Uppsala.

Fahlvik, N., B. Elfving, and W. P. 2014. Evaluation of growth functions used in the Swedish Forest Planning System Heureka. Silva Fennica **48**: article id 101.

Gourbiere, S., and F. Gourbiere. 2002. Competition between unit-restricted fungi: A metapopulation model. Journal of Theoretical Biology **217**:351-368.

Gu, W. D., R. Heikkilä, and I. Hanski. 2002. Estimating the consequences of habitat fragmentation on extinction risk in dynamic landscapes. Landscape Ecology **17**:699-710.

Halme, P., and J. S. Kotiaho. 2012. The importance of timing and number of surveys in fungal biodiversity research. Biodiversity and Conservation **21**:205-219.

Harmon, M. E., O. N. Krankina, and J. Sexton. 2000. Decomposition vectors: a new approach to estimating woody detritus decomposition dynamics. Canadian Journal of Forest Research **30**:76-84.

Harrison, P. J., I. Hanski, and O. Ovaskainen. 2011. Bayesian state-space modeling of metapopulation dynamics in the Glanville fritillary butterfly. Ecological Monographs **81**:581-598.

Heilmann-Clausen, J., and L. Boddy. 2008. Distribution patterns of wood-decay basidiomycetes at the landscape to global scale. Pages 263-275 *in* L. Boddy, J. C. Frankland, and P. van West, editors. Ecology of Saprotrophic Basidiomycetes. Elsevier, Amsterdam.

Hynynen, J., H. Salminen, A. Ahtikoski, S. Huuskonen, R. Ojansuu, J. Siipilehto, M. Lehtonen, A. Rummukainen, S. Kojola, and K. Eerikäinen. 2014. Analysis for the Biomass Supply Potential and the Future Development of Finnish Forest Resources.

Ingemarson, F., A. Lindhagen, and L. Eriksson. 2006. A typology of small-scale private forest owners in Sweden. Scandinavian Journal of Forest Research **21**:249-259.

Jonsson, B. G. 2000. Availability of coarse woody debris in a boreal old-growth Picea abies forest. Journal of Vegetation Science **11**:51-56.

Jonsson, B. G., M. Ekström, P. A. Esseen, A. Grafström, G. Ståhl, and B. Westerlund. 2016. Dead wood availability in managed Swedish forests - Policy outcomes and implications for biodiversity. Forest Ecology and Management **376**:174-182.

Juutilainen, K., P. Halme, H. Kotiranta, and M. Mönkkönen. 2011. Size matters in studies of dead wood and wood-inhabiting fungi. Fungal Ecology **4**:342-349.

Jönsson, M. T., M. Edman, and B. G. Jonsson. 2008. Colonization and extinction patterns of wood-decaying fungi in a boreal old-growth Picea abies forest. Journal of Ecology **96**:1065-1075.

Kéry, M., J. H. Spillmann, C. Truong, and R. Holderegger. 2006. How biased are estimates of extinction probability in revisitation studies? Journal of Ecology **94**:980-986.

MacKenzie, D. I., J. D. Nichols, J. E. Hines, M. G. Knutson, and A. B. Franklin. 2003. Estimating site occupancy, colonization, and local extinction when a species is detected imperfectly. Ecology **84**:2200-2207.

Mair, L., P. J. Harrison, M. Jönsson, S. Löbel, J. Nordén, J. Siitonen, T. Lämås, A. Lundström, and T. Snäll. 2017. Evaluating citizen science data for forecasting species responses to national forest management. Ecology and Evolution **7**:368-378.

Mair, L., M. Jönsson, M. Räty, L. Bärring, G. Strandberg, T. Lämås, and T. Snäll. 2018. Land use changes could modify future negative effects of climate change on old-growth forest indicator species. Diversity and Distributions **24**:1416-1425.

Nordén, J., R. Penttilä, J. Siitonen, E. Tomppo, and O. Ovaskainen. 2013. Specialist species of wood-inhabiting fungi struggle while generalists thrive in fragmented boreal forests. Journal of Ecology **101**:701-712.

Nordén, J., J. Åström, T. Josefsson, S. Blumentrath, O. Ovaskainen, A. Sverdrup-Thygeson, and B. Nordén. 2018. At which spatial and temporal scales can fungi indicate habitat connectivity? Ecological Indicators **91**:138-148.

Norros, V., R. Penttilä, M. Suominen, and O. Ovaskainen. 2012. Dispersal may limit the occurrence of specialist wood decay fungi already at small spatial scales. Oikos **121**:961-974.

Ovaskainen, O., D. Schigel, H. Ali-Kovero, P. Auvinen, L. Paulin, B. Nordén, and J. Nordén. 2013. Combining high-throughput sequencing with fruit body surveys reveals contrasting life-history strategies in fungi. Isme Journal **7**:1696-1709.

Paltto, H., B. Nordén, F. Götmark, and N. Franc. 2006. At which spatial and temporal scales does landscape context affect local density of Red Data Book and Indicator species? Biological Conservation **133**:442-454.

Rhodes, J. R., A. J. Tyre, N. Jonzen, C. A. McAlpine, and H. P. Possingham. 2006. Optimizing presence-absence surveys for detecting population trends. Journal of Wildlife Management **70**:8-18.

Ruete, A., T. Snäll, B. G. Jonsson, and M. Jönsson. 2017. Contrasting long-term effects of transient anthropogenic edges and forest fragment size on generalist and specialist deadwood-dwelling fungi. Journal of Applied Ecology **54**:1142-1151.

Salminen, H., M. Lehtonen, and J. Hynynen. 2005. Reusing legacy FORTRAN in the MOTTI growth and yield simulator. Computers and Electronics in Agriculture **49**:103-113.

Sandström, F., H. Petersson, N. Kruys, and G. Ståhl. 2007. Biomass conversion factors (density and carbon concentration) by decay classes for dead wood of Pinus sylvestris, Picea abies and Betula spp. in boreal forests of Sweden. Forest Ecology and Management **243**:19-27.

Snäll, T., J. Pennanen, L. Kivisto, and I. Hanski. 2005. Modelling epiphyte metapopulation dynamics in a dynamic forest landscape. Oikos **109**:209-222.

Sverdrup-Thygeson, A., L. Gustafsson, and J. Kouki. 2014. Spatial and temporal scales relevant for conservation of dead-wood associated species: current status and perspectives. Biodiversity and Conservation **23**:513-535.

Tomppo, E., M. Haakana, M. Katila, and J. Peräsaari. 2008. Multi-source national forest inventory - Methods and applications. Springer, Dordrecht.

Wikberg, P.-E. 2004. Occurrence, morphology and growth of understory saplings in Swedish forests. Acta Universitatis agriculturae Sueciae.

Wikström, P., L. Edenius, B. Elfving, L. O. Eriksson, T. Lämås, J. Sonesson, K. Öhman, J. Wallerman, C. Waller, and F. Klintebäck. 2011. The Heureka Forestry Decision Support System: An Overview. 2011 **3**.
